# Secreted microvesicular miR‐31 inhibits osteogenic differentiation of mesenchymal stem cells

**DOI:** 10.1111/acel.12484

**Published:** 2016-05-04

**Authors:** Sylvia Weilner, Elisabeth Schraml, Matthias Wieser, Paul Messner, Karl Schneider, Klemens Wassermann, Lucia Micutkova, Klaus Fortschegger, Andrea B. Maier, Rudi Westendorp, Heinrich Resch, Susanne Wolbank, Heinz Redl, Pidder Jansen‐Dürr, Peter Pietschmann, Regina Grillari‐Voglauer, Johannes Grillari

**Affiliations:** ^1^ Department of BiotechnologyBOKU ‐ University of Natural Resources and Life Sciences ViennaMuthgasse 181190ViennaAustria; ^2^ Ludwig Boltzmann Institute for Experimental and Clinical TraumatologyAUVA Research CenterDonaueschingenstrasse 13A‐1200ViennaAustria; ^3^ Evercyte GmbHMuthgasse 181190ViennaAustria; ^4^ ACIBMuthgasse 181190ViennaAustria; ^5^ Department of NanoBiotechnologyVienna Institute of BioTechnologyUniversity of Natural Resources and Life Sciences ViennaViennaAustria; ^6^ Institute of Biomedical Aging ResearchAustrian Academy of SciencesViennaAustria; ^7^ Children's Cancer Research Institute (CCRI)St. Anna KinderkrebsforschungViennaAustria; ^8^ Department of Medicine and Aged CareRoyal Melbourne HospitalUniversity of MelbourneMelbourneAustralia; ^9^ Department of Human Movement SciencesMOVE Research Institute AmsterdamVrije Universiteit AmsterdamAmsterdamThe Netherlands; ^10^ Department of public health and center for healthy aginguniversity of CopenhagenDenmark; ^11^ Department of Medicine 2St. Vincent Hospital1060ViennaAustria; ^12^ Department of Pathophysiology and Allergy ResearchCenter of PathophysiologyInfectiology and ImmunologyMedical University of Vienna1090ViennaAustria; ^13^ Austrian Cluster for Tissue RegenerationViennaAustria

**Keywords:** aging, mesenchymal stem cells, MicroRNA, osteogenic differentiation, microvesicles, senescence‐associated secretory phenotype

## Abstract

Damage to cells and tissues is one of the driving forces of aging and age‐related diseases. Various repair systems are in place to counteract this functional decline. In particular, the property of adult stem cells to self‐renew and differentiate is essential for tissue homeostasis and regeneration. However, their functionality declines with age (Rando, 2006). One organ that is notably affected by the reduced differentiation capacity of stem cells with age is the skeleton. Here, we found that circulating microvesicles impact on the osteogenic differentiation capacity of mesenchymal stem cells in a donor‐age‐dependent way. While searching for factors mediating the inhibitory effect of elderly derived microvesicles on osteogenesis, we identified miR‐31 as a crucial component. We demonstrated that miR‐31 is present at elevated levels in the plasma of elderly and of osteoporosis patients. As a potential source of its secretion, we identified senescent endothelial cells, which are known to increase during aging *in vivo* (Erusalimsky, 2009). Endothelial miR‐31 is secreted within senescent cell‐derived microvesicles and taken up by mesenchymal stem cells where it inhibits osteogenic differentiation by knocking down its target Frizzled‐3. Therefore, we suggest that microvesicular miR‐31 in the plasma of elderly might play a role in the pathogenesis of age‐related impaired bone formation and that miR‐31 might be a valuable plasma‐based biomarker for aging and for a systemic environment that does not favor cell‐based therapies whenever osteogenesis is a limiting factor.

## Introduction

Various repair systems at the tissue level are at work to counteract the functional decline driving and accompanying the organismal aging process, such as adult stem cells (Liu & Rando, 2011). However, also stem cell functionality declines with age due to both intrinsic and extrinsic factors (Liu & Rando, 2011). In particular, the systemic environment of the elderly has been shown to negatively impact on them, either by circulating factors that inhibit stem cell functions, or by the absence of factors that would be needed to support it (Conboy *et al*., 2005; Matsumoto *et al*., 2009). However, such factors, sources, and molecular mechanisms involved are not well understood yet. Consequently, gaining insights into the aging systemic environment and its impact on stem cell regulation might be a key for the successful application of cell‐based therapies for the elderly. As senescent human cells are known to have an altered secretory behavior, termed senescence‐associated secretory phenotype (SASP), such extrinsic factors that inhibit tissue repair and regeneration might be provided by senescent cells. Indeed, the importance of senescence *in vivo* was demonstrated recently, as removal of senescent cells postpones the onset of age‐associated diseases (Baker *et al*., 2011), similar to the anti‐aging effects obtained by re‐elongation of eroded telomeres (Jaskelioff *et al*., 2011).

Specifically, endothelial cells (ECs), which line the blood vessels, are in close proximity to mesenchymal stem cells, and senescent ECs are known to accumulate in the vessel wall with age (Minamino & Komuro, 2007; Erusalimsky, 2009). This suggests that senescent endothelial cells may secrete specific factors impacting on the systemic as well as local environment of mesenchymal stem cells during aging. Unlike SASP of human fibroblasts (Coppé *et al*., 2008), very little data are yet available on the endothelial SASP (eSASP).

Microvesicles were recently described among cellularly secreted factors. They were not only shown to be secreted by almost all cell types *in vitro,* but also isolated from biological fluids like blood (Mathivanan *et al*., 2010). These vesicles do contain proteins, as well as mRNAs and noncoding RNAs and were demonstrated to load their cargo specifically (Mathivanan *et al*., 2010). Upon secretion, these vesicles are able to transport their cargo over short and long distances, to protect it from degradation and to specifically deliver it to target cells (Mathivanan *et al*., 2010). Therefore, microvesicles are thought to pose a new way of cell‐to‐cell communication.

In this study, we investigated the effect of senescent compared with early passage endothelial cell‐derived microvesicles, as well as of microvesicles isolated from the plasma of elderly individuals compared with vesicles from young donors on *in vitro* bone formation.

## Results

**Figure 1:**
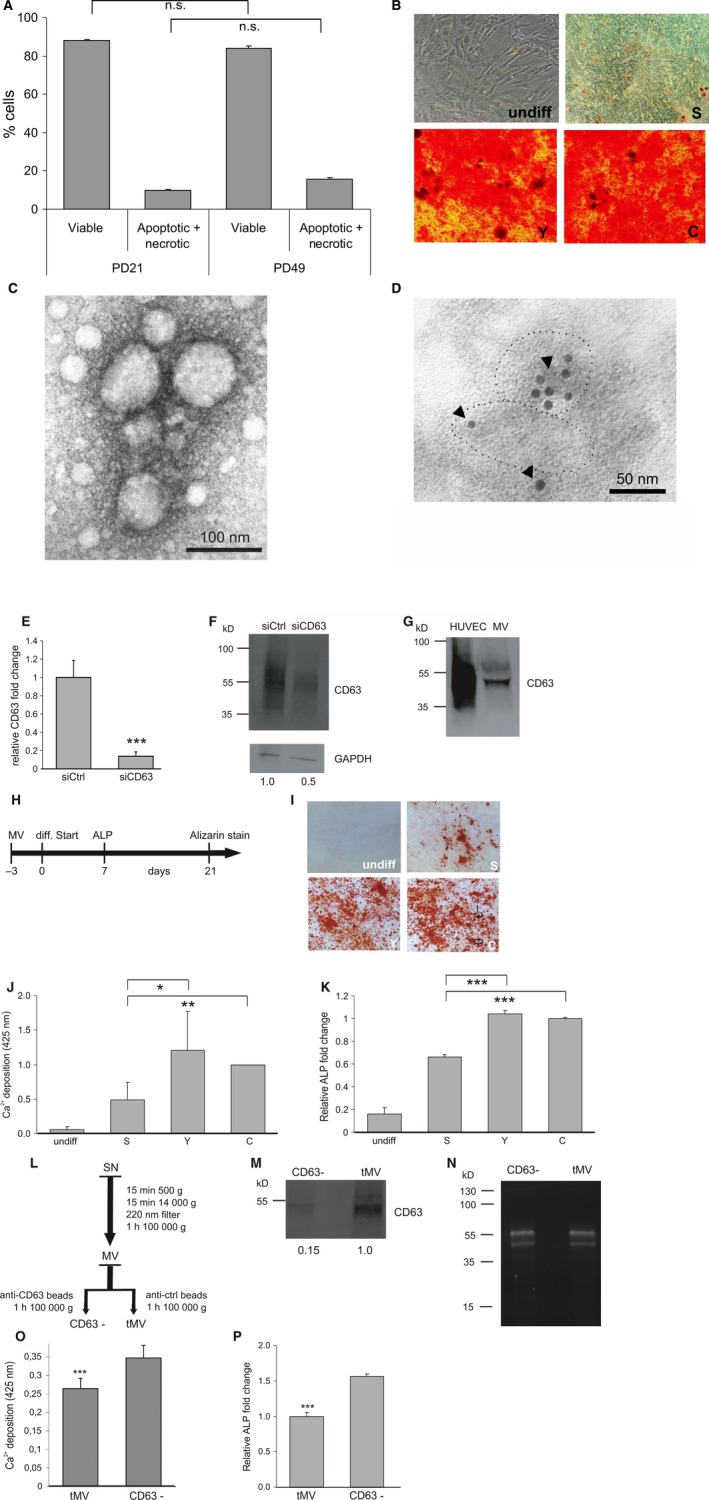
CD63‐positive microvesicles (MVs) of senescent HUVECs reduce osteogenic differentiation capacity of ASCs. (A) Apoptotic cell death of early quiescent passage (PD21) or replicative senescent (PD49) HUVECs 48 h after secretion into ASC medium (*N *= 3) (B) Representative picture of Alizarin Red S staining for Ca^2+^ deposition of ASCs induced to undergo osteogenic differentiation and exposed to conditioned media of early quiescent passage (Y), senescent (S) or unconditioned medium (C) as well as without switching to differentiation medium (undiff). (C) EM image of MVs isolated from conditioned medium of HUVECs. The EM image shows representative MV membrane vesicles of ~ 50–100 nm in diameter. (D) CD63 as marker of MVs was immunogold‐labeled with anti‐CD63 antibody (5‐nm gold particles, see arrows) and visualized by electron microscopy. The dotted lines accentuate the border of selected MVs adsorbed to the coated Ni‐grid. (E) qPCR showing decreased intracellular CD63 levels 48 h after transient transfection of ASCs with siRNA against CD63 (siCD63) compared with nontargeting siRNA control (siCtrl)‐transfected cells. (F) CD63 protein levels 48 h after transient transfection of ASCs with siRNA against CD63 (siCD63) are reduced compared with nontargeting siRNA control (siCtrl)‐transfected cells when normalized to GAPDH. (G) Western blot analysis of HUVEC cell lysate and endothelial microvesicles (MV) using anti‐CD63 antibody. (H) Time scheme of experimental design. ASCs were exposed to MVs from early quiescent passage (13 PD) or senescent (53PD) cells and unconditioned control medium for 3 days. Then, medium was changed to differentiation medium. Alizarin Red S staining for Ca^2+^ deposits was performed after 21 days. In addition, qPCR for the osteogenic mRNA marker alkaline phosphatase (ALP) was performed after 7 days. (I–K) ASCs were pre‐incubated in the presence of MVs derived from early quiescent passage (Y), senescent (S) cells or in the absence of exogenously added MVs (C) before osteogenic differentiation was induced as well as without switching to differentiation medium (undiff). (I) Representative images and (J) quantification of calcium depositions are shown. (K) qPCR for ALP mRNA was performed. Error bars indicate the standard deviations of 3 independent measurements. (L) Scheme of experimental design. After 48 h of secretion, conditioned supernatant (SN) was collected from senescent cells and MVs were isolated by differential centrifugation as described in the chapter ‘experimental procedures’. Subsequently, the MV containing pellet was resuspended in 2 mL PBS. One milliliter was loaded for 2 h at 4°C on anti‐CD63 antibody‐coupled magnetic beads, while the remaining 1 mL was exposed to magnetic beads coupled to IgG control antibody. After 2 h of incubation, tMV as well as the supernatant of the immune isolated fraction containing no CD63‐positive vesicles, referred to as CD63^−^ fraction (CD63^−^), was centrifuged for 1 h at 100 000 g. Finally, the pellets of the tMV and of the CD63^−^ fraction were resuspended in PBS and added to ASCs. (M) Western blot analysis of the tMV fraction and the CD63^−^ fraction using anti‐CD63 antibody. (N) Image of the corresponding gel before blotting showing proteins stained with trihalo compounds as a loading control. (O–P) Decreased osteogenic differentiation potential of ASCs exposed to isolated tMV fraction of senescent endothelial cells compared with ASCs treated with CD63^−^ fraction as quantified by (O) Alizarin Red S staining and (P) the early osteogenic differentiation marker ALP using qPCR. ns: not significant, *: *P *< 0.05, **: *P* < 0.01, ***: *P* < 0.001 in comparison with control. Data are presented as mean values ± SD and were statistically analyzed using Student's *t*‐test.

To address the possibility that components of the eSASP would influence mesenchymal stem cell (MSC) function, adipose tissue‐derived MSCs (ASCs) were exposed to conditioned medium derived from senescent or quiescent early passage human umbilical vein endothelial cells (HUVECs) for 3 days before inducing osteogenic differentiation. Alizarin Red S staining for Ca^2+^ deposition was performed 21 days after induction. ASCs, which were isolated from different donors, showed typical morphology (Fig. S1A) and stained positive for adult mesenchymal as well as negative for typical hematopoietic stem cell marker (Fig. S1B). Senescent endothelial cells were growth‐arrested, showed a large flattened morphology, and stained positive for SA‐β‐gal activity (Fig. S1C) as published earlier (Chang *et al*., 2005). No significant increase in apoptosis was observed during the 48‐h period of secretion under all experimental conditions (Fig. 1A), thereby excluding effects mediated by apoptotic vesicles. Senescent endothelial cell supernatants decreased the osteogenic differentiation of ASCs (Fig. 1B) compared with ASCs exposed to conditioned medium of early passage HUVECs, as visualized by staining for Ca^2+^ deposition using Alizarin Red S staining.

Recently, microvesicles (MVs) were discovered to be secreted *in vitro* by various cell types but also to be present in body fluids, such as blood, as reviewed by Mathivanan *et al*. (2010). They serve as vehicles for RNA and proteins and enable a protected and targeted transport from the donor to the recipient cell, thereby representing a new type of cell–cell communication (Mathivanan *et al*., 2010). We isolated the fraction containing microvesicles below 220 nm in size by differential centrifugation (Deregibus *et al*., 2007), hereinafter referred to as MV fraction, from cell culture supernatant of quiescent, early passage HUVECs and of replicatively senescent HUVECs in order to narrow down the fraction of supernatant containing the osteogenesis inhibitory activity. Electron microscopy confirmed the isolation of membrane vesicles, which were smaller than 100 nm in size (Fig. 1C), and reacted positive for CD63 staining (Fig. 1D), a transmembrane protein and established microvesicular marker (Valadi *et al*., 2007).

In addition, we aimed to check the presence of CD63‐positive MVs in the MV fraction isolated from conditioned medium by Western blot. Therefore, antibody specificity was tested by comparing the amount of CD63 protein in HUVECs transfected with siRNA against CD63 or the corresponding control. Successful knockdown of CD63 mRNA was confirmed by qPCR (Fig. 1E), and protein lysates isolated from siRNA against CD63‐transfected cells showed a reduced signal for CD63 compared with control‐transfected cells when analyzed by Western blot (Fig. 1F). Finally, we analyzed proteins isolated from the obtained MV fraction and from HUVEC‐derived cell lysates as positive control. In both cases, a strong signal for CD63 was observed (Fig. 1G), confirming that micorvesicles positive for CD63 were successfully isolated.

Next, ASCs were exposed to MV fraction isolated from conditioned media of senescent or early passage HUVECs for 3 days before osteogenesis was induced (Fig. 1H). Indeed, osteogenic differentiation of ASCs incubated with MVs of senescent cells was significantly decreased, as shown by a reduction of calcium deposition (Fig. 1I,J) and by decreased expression of the osteogenic marker gene alkaline phosphatase (ALP) (Fig. 1K) compared with ASCs exposed to MVs secreted by quiescent early passage endothelial cells.

To confirm that the osteogenesis inhibitory activity of the total MV fraction indeed resides in the MVs and not in protein aggregates that might cosediment, we depleted the pellet fraction after ultracentrifugation from CD63‐positive MVs by immuno‐affinity capture (Fig. 1L) using anti‐CD63 antibody‐coupled beads, hereinafter referred to as CD63^‐^ fraction. An image of the SDS–PAGE gel loaded with MVs exposed to CD63 or control IgG antibody‐coupled beads and stained with trihalo compounds before Western blotting confirms that equal amounts of proteins were loaded (1M). Successful reduction of CD63‐positive vesicles from the MV fraction, compared with MVs exposed to IgG control antibody‐coupled beads, was confirmed by Western blot (Fig. 1N).

To control whether the depletion of the CD63^+^ microvesicles also rescues the inhibitory effect of the MVs isolated from senescent HUVECs, ASCs were exposed to the total MV fraction, or the CD63^−^ fraction, as outlined in Fig. 1L. Subsequently, osteogenic differentiation was induced and quantified by changes in Ca^2+^ deposition ability and ALP mRNA. Indeed, the inhibitory activity of the senescent cell‐derived total MV fraction was depleted with the depletion of CD63‐positive microvesicles (Fig. 1O,P), indicating that the inhibitory activity is mediated by senescent HUVEC‐derived CD63‐positive MVs.

**Figure 2:**
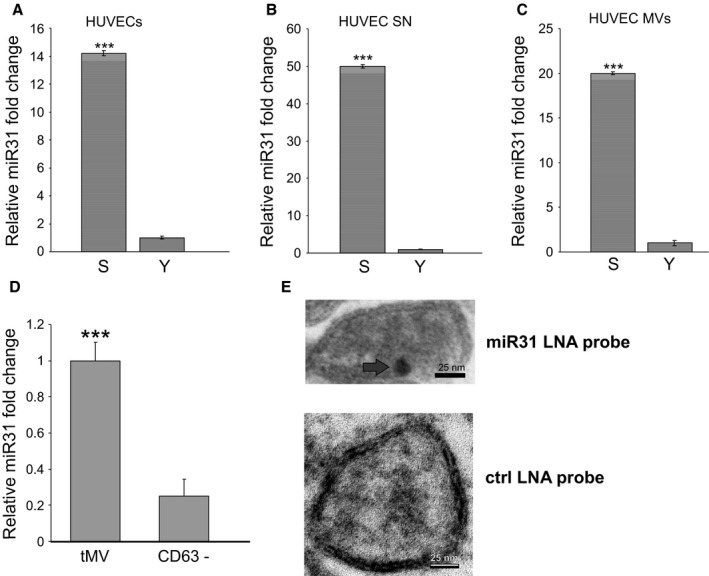
miR‐31 is enriched in CD63‐positive microvesicles of senescent endothelial cells. (A) miR‐31 expression is upregulated in senescent (S) versus early quiescent passage (Y) HUVECs (*N *= 3) as analyzed by qPCR. (B) miR‐31 was detected at high levels after RNA isolation from supernatants (SN) (*N *= 3) and (C) from MVs of senescent (S) versus early quiescent (Y) passage HUVECs (*N *= 3). (D) Significantly increased miR‐31 levels in tMV fraction of senescent endothelial cells compared with the CD63^−^ fraction. (E) Localization of miR‐31 within MVs. Arrowhead indicates immunogold‐labeled anti‐DIG antibody after hybridizing a DIG‐labeled anti‐miR‐31 probe to permeabilized MVs. ***: *P* < 0.001 in comparison with control. Data of this figure are presented as mean values ± SD and were statistically analyzed using Student's *t*‐test.

**Figure 3:**
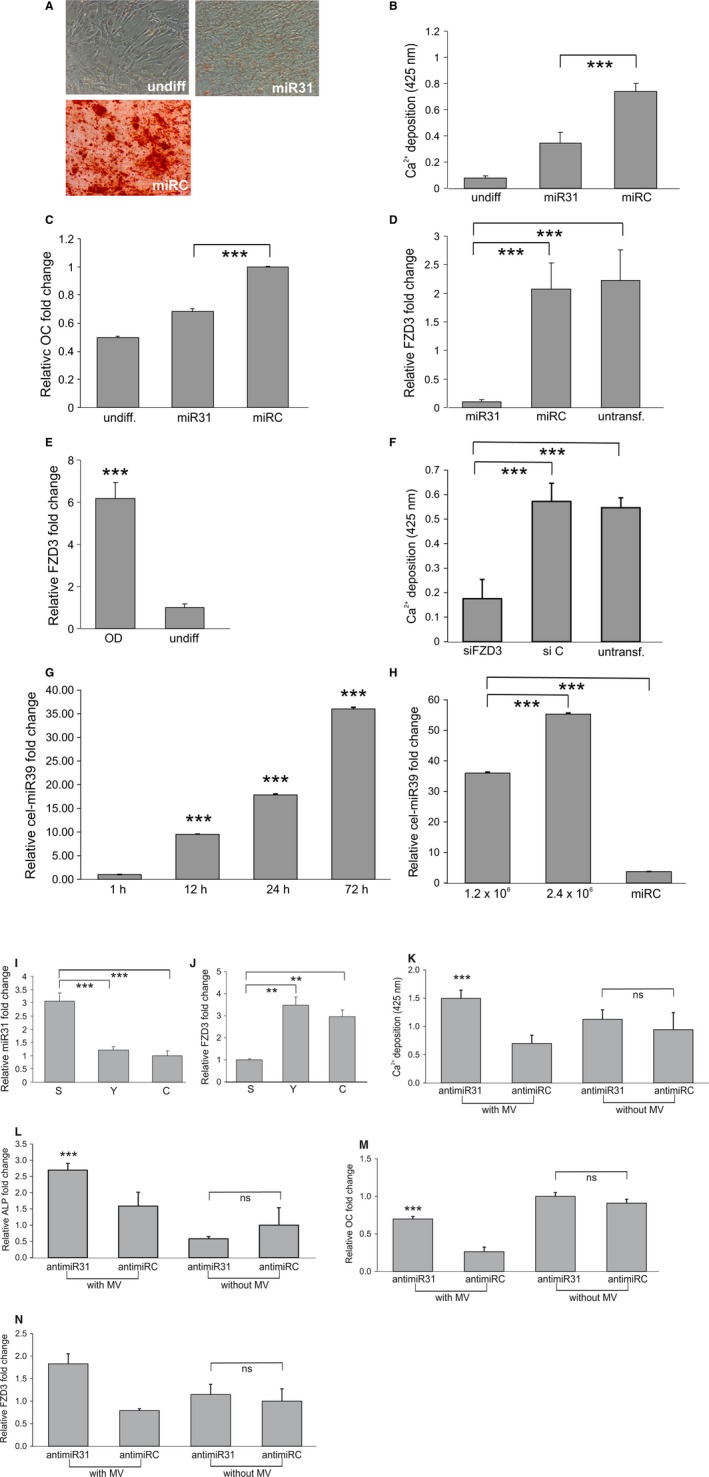
Vesicular miR‐31 reduces osteogenesis by knocking down FZD3 mRNA. (A–C) ASCs were transfected with miR‐31 or a nontargeting miRNA control (miRC) before inducing osteogenesis. As control, ASCs were not exposed to differentiation inducing medium (undiff). (A) Representative images and (B) quantification of calcium depositions as well as (C) qPCR for the osteogenic marker OC confirm inhibition of osteogenic differentiation after miR‐31 transfection. (D) 4 days after induction of osteogenesis miR‐31 transfection of ASCs results in downregulated, FDZ3 mRNA compared with nontargeting control‐transfected cells (miRC) and nontransfected (untransf) cells as quantified by qPCR. (E) FDZ3 mRNA is upregulated 4 days after osteogenic differentiation start (OD) as compared to undifferentiated ASCs (undiff) as analyzed by qPCR. (F) Knockdown of FDZ3 by siRNAs (siFZD3) in ASCs inhibits osteogenic differentiation compared with nontargeting control‐transfected (siC) or untransfected (untransf.) cells as quantified by Alizarin Red S staining. (G) Intracellular cel‐miR‐39 levels of ASCs that were exposed to MVs isolated from cel‐miR‐39‐transfected HUVECs for 1, 12, 24, or 72 h were quantified by qPCR. (H) Intracellular cel‐miR‐39 levels of ASCs that were exposed for 72 hours to MVs isolated from the indicated number of cel‐miR‐39‐transfected HUVECs or from nontargeting control (miRC)‐transfected HUVECs were quantified by qPCR. Intracellular levels of miR‐31 (I) and FZD3 mRNA (J) in ASCs after treatment with MVs isolated from early quiescent passage (Y), senescent (S) HUVECs or unconditioned medium (C), were quantified by qPCR. (K–M) ASCs were transfected with anti‐miR‐31 or a nontargeting miRNA control (anti‐miRC). Twenty‐four hours after transfection, either no or MVs of senescent HUVECs were added. (K) Quantification of calcium depositions, (L) ALP mRNA levels, (M) OC mRNA levels, and (N) FZD3 mRNA levels reveal an increased differentiation capacity of MV‐treated ASCs upon anti‐miR‐31 transfection. Error bars derived from 3 independent experiments. ND: nondifferentiated. ns: not significant, **: *P* < 0.01, ***: *P* < 0.001 in comparison with control. Data are presented as mean values ± SD and were statistically analyzed using Student's *t*‐test.

As miRNAs have been reported to be packaged into MVs (Hunter *et al*., 2008), we analyzed the expression of age‐related miRNA species (Hackl *et al*., 2010) in MVs derived from early quiescent versus senescent cells and focused on miR‐31 because it is upregulated intracellularly in senescent HUVECs (Hackl *et al*., 2010) and because it recently turned out to be a master regulator of osteogenesis by targeting Runx2 (Deng *et al*., 2013a, 2013b), Osterix (Baglio *et al*., 2013), and SATB2 (Deng *et al*., 2013a, 2013b). We confirmed that miR‐31 is upregulated in HUVECs during both replicative (Fig. 2A) and stress‐induced senescence (Fig. S2A–C), as well as in senescent human liver endothelial cells (Fig. S2D) and human retinal microvascular endothelial cells (Fig. S2E) compared with corresponding quiescent early passage cells. In addition, miR‐31 expression was increased in conditioned medium (Fig. 2B) and in MVs (Fig. 2C) derived from replicatively senescent (Fig. 2B,C) compared with quiescent, early passage cells. In particular, microvesicular miR‐31 was found to be enriched in the total MV fraction (~fourfold) (Fig. 2D) compared with the CD63^−^ fraction isolated from senescent endothelial cells, suggesting that the majority of endothelially secreted miR‐31 is secreted within CD63‐ positive MVs. Its localization within MVs was additionally confirmed by electron microscopy of in situ hybridized samples (Fig. 2E). To test whether miR‐31 alone is sufficient to inhibit osteogenic differentiation of ASCs, we then proceeded to transiently transfect ASCs with miR‐31 mimics. Transfection success was confirmed by qPCR (Fig. S3A). Indeed, miR‐31 alone was sufficient to significantly inhibit osteogenic differentiation by ~50% as visualized and quantitated by Alizarin Red S staining (Fig. 3A,B), as well as by decreased expression of osteocalcin (OC) mRNA (Fig. 3C). This is supported by our findings that miR‐31 overexpression in both C3Ht101/2 and C2C12 cells, representing two established mouse model systems of osteogenic differentiation (Feichtinger *et al*., 2011), resulted in an anti‐osteogenic effect as well (Fig. S3B, C).

MiR‐31 regulates FZD3, a Wnt5A receptor, in the context of breast cancer invasiveness (Valastyan *et al*., 2009), and we observed that FZD3 mRNA was also downregulated upon miR‐31 transfection in ASCs (Fig. 3D). To test whether FZD3 has a crucial role in osteogenesis, we compared FZD3 mRNA levels of differentiated versus undifferentiated ASCs and found that FZD3 mRNA is upregulated during osteogenesis at day 4 compared with nondifferentiating ASCs (Fig. 3E). To address the question whether the depletion of FZD3 mRNA would be sufficient to inhibit osteogenesis, FZD3 gene expression was silenced by specific siRNA (Fig. S3D), which led to a significant inhibition of osteogenesis compared with nontargeting siRNA‐transfected cells (Fig. 3F).

To test whether endothelial‐derived MVs are able to interact with and to release their content into ASCs and thus transfer microRNAs from endothelial cells to ASCs, HUVECs were transfected with cel‐miR‐39, a *Caenorhabditis elegans* miRNA with no homolog in humans (Hergenreider *et al*., 2012). MVs isolated from transfected HUVECs were added to ASCs, and indeed, intracellular cel‐miR‐39 levels of ASCs increased with exposure time (Fig. 3G) and microvesicular dose (Fig. 3H).

To see whether miR‐31 might be transferred to ASCs as recipient cells via MVs, we exposed ASCs to senescent or early passage cell‐derived MVs. Indeed, exposure to senescent cell‐derived microvesicles resulted in a threefold increase in miR‐31 levels in ASCs (Fig. 3I) compared with ASCs exposed to quiescent early passage HUVECs, accompanied by a decrease in FZD3 mRNA (Fig. 3J).

To test whether microvesicularly transferred miR‐31 is crucial to confer the inhibitory effect of senescent HUVEC‐derived MVs on osteogenic differentiation, we pretransfected ASCs, using anti‐miR‐31 or a nontargeting anti‐miRNA as control, 24 h before exposing them to the MV fraction isolated from conditioned medium of senescent HUVECs. Transfection success of anti‐miR‐31 was confirmed (Fig. S3E) by qPCR. Anti‐miR‐31 transfection indeed rescued the inhibition of osteogenic differentiation, mediated by MVs of senescent HUVECs, as quantified by Alizarin Red S staining (Fig. 3K) and qPCR on ALP (Fig. 3L) and OC mRNA (Fig. 3M). Moreover, anti‐miR‐31 transfection of ASCs also rescued the decrease in FZD3 mRNA levels upon treatment of ASCs with MVs (Fig. 3N). To exclude the possibility that the observed restored osteogenic differentiation capacity results from the anti‐miR‐31 transfection, we transfected ASCs with anti‐miR‐31 or a nontargeting anti‐miRNA as control without exposing them to MVs. Anti‐miR‐31 transfection of ASCs alone had no significant influence on osteogenic differentiation capacity as shown by Alizarin Red S staining (Fig. 3K), on ALP (Fig. 3L), OC (Fig. 3M), and FZD3 (Fig. 3M) mRNA levels.

**Figure 4:**
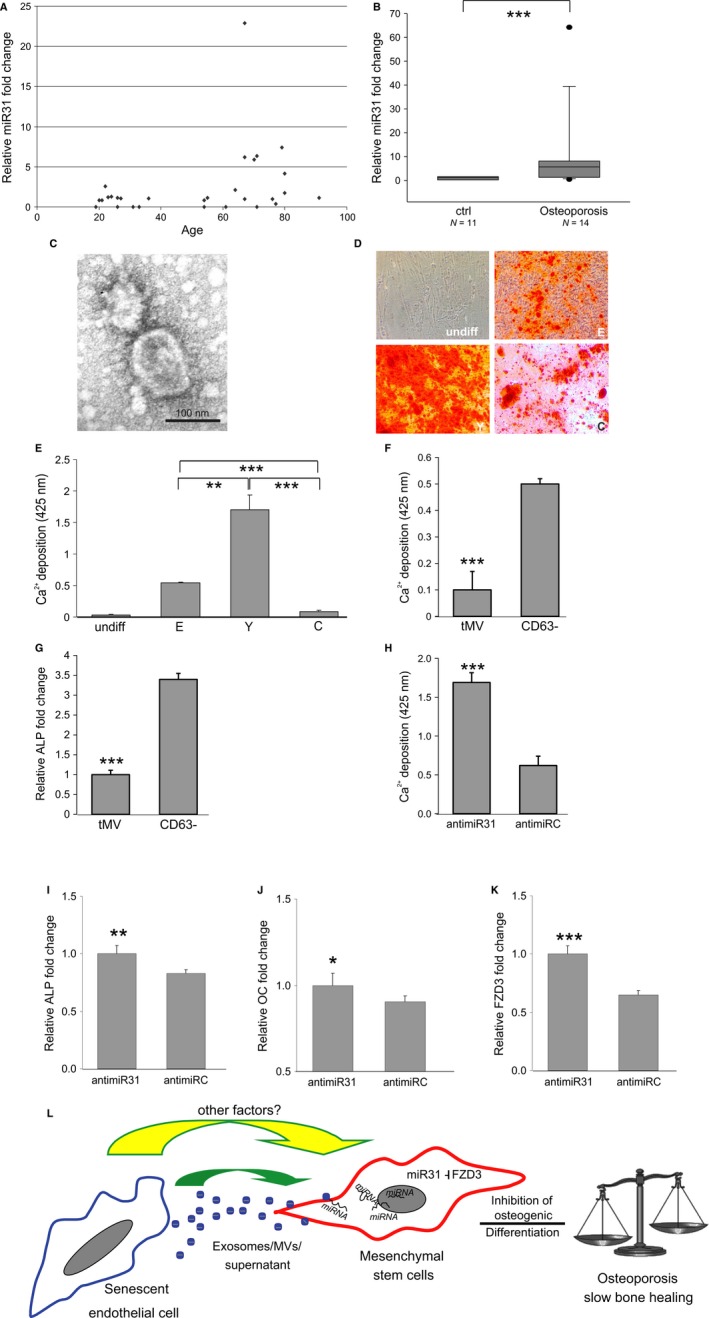
Effects of plasma MVs derived from young and elderly human donors on ASCs. (A) Upregulation of miR‐31 levels in plasma samples derived from healthy, female elderly donors (*n *= 17) compared with young healthy controls (*n *= 12). (B) miR‐31 levels in plasma derived from male osteoporosis patients (*n *= 14) were significantly increased compared with healthy age‐matched controls (*n *= 11). (C) MVs showing typical morphology as analyzed by electron microscopy. (D–E) ASCs were exposed to MVs derived from healthy donors younger than 25 (Y) and elderly donors of above 55 years (E). As controls, no MVs were added (C) as well as no differentiation was induced (undiff). (D) Representative images and (E) quantification of Alizarin Red S stained cells after differentiation. Results derived from 3 independent replicates of each donor are shown. (F–G) ASCs showed a decreased osteogenic differentiation potential after treatment with the total MV fraction (tMV) compared with ASCs exposed to the CD63‐negative fraction (CD63^−^) as (F) quantified by alizarin red S staining for Ca^2+^ deposition and (G) shown on the basis of ALP mRNA. (H–J) Anti‐miR‐31 or nontargeting miRNA control (anti‐miRC)‐transfected ASCs were exposed to total MV fraction from an elderly miR‐31 high donor. (H) Osteogenic differentiation was analyzed by quantitation of calcium depositions and by qPCR on (I) ALP, (J) OC, and (K) FZD3 mRNA levels. Analysis showing an increase in osteogenic differentiation capacity of ASCs co‐incubated with MVs upon anti‐miR‐31 transfection. (L) Overview of our working hypothesis: Exosomes/MVs/Supernatant derived from senescent endothelial cells affect differentiation potential of ASCs via miRNA‐31 delivery by knocking down its target FZD3 mRNA. Therefore, the ‘senescent’ environment hampers tissue regeneration by inhibiting the induction of osteogenesis. Therefore, the systemic environment of the elderly might favor loss of bone mass and inhibit bone healing. *: *P* < 0.05, **: *P* < 0.01, ***: *P* < 0.001 in comparison with control. Data are presented as mean values ± SD and were statistically analyzed using Wilcoxon signed rank test in Fig. 4B and Student's *t*‐test for Fig. 4E–K.

As senescent cells accumulate with age *in vivo* and as endothelial cells line the vasculature, we were curious to see whether miR‐31 might also be secreted to the circulation. We did not observe large differences in miR‐31 levels in plasma of young donors. However, we did observe large variations and a subpopulation with significantly increased miR‐31 plasma levels in the group of women older than 50 years (Fig. 4A).

We reasoned that if high miR‐31 is related to decreased bone formation, we might expect upregulated plasma miR‐31 levels also in osteoporotic patients. Indeed, miR‐31 levels in plasma derived from male osteoporosis patients were significantly increased compared with healthy age‐matched controls (Fig. 4B).

Based on these results, we addressed the question whether microvesicles isolated from elderly individuals with high miR‐31 levels in the plasma would also inhibit osteogenesis. Therefore, we isolated MVs from human blood plasma and performed electron microscopy to confirm size and shape (Fig. 4C). Indeed, total MV fraction derived from elderly donors did not facilitate osteogenic differentiation compared with MVs isolated from plasma of young individuals (Fig. 4D and E). To test whether the inhibitory activity of the total MV fraction isolated from elderly donors again resides in the CD63‐positive MVs, we depleted them from the total MV fraction by affinity purification as previously outlined in Fig. 1L. Co‐incubation of ASCs with the CD63^−^ fraction of an elderly miR‐31 high donor resulted in an enhanced osteogenic differentiation capacity compared with ASCs exposed to the total MV fraction as confirmed by quantification of Ca^2+^ depositions (Fig. 4F) and qPCR of ALP mRNA (Fig. 4G), indicating that the inhibitory activity again resided in the CD63‐positive MV fraction.

Finally, we confirmed that even in the case of *ex vivo*‐derived MVs of elderly donors, miR‐31 is a crucial osteogenesis‐impacting microvesicular factor, as anti‐miR‐31 transfection of ASCs, prior to MV fraction exposure, rescued the inhibitory effect of plasma‐derived MVs as quantified by Alizarin Red S staining (Fig. 4H), ALP (Fig. 4I), as well as OC (Fig. 4J) mRNA levels. In addition, FZD3 mRNA levels were restored (Fig. 4K).

## Discussion

Here, we set out to identify factors that contribute to the negative effect of the aged systemic environment on the functionality of adult stem cells (Rando, 2006). With regard to bone and its regeneration capacity, mesenchymal stem cells give rise to bone‐forming osteoblasts, but there is evidence that their osteogenic differentiation capacity decreases with age (Roholl *et al*., 1994; Zhu *et al*., 2009). Therefore, we focused on extracellular factors impacting on osteogenic differentiation capacity of ASCs. Besides well‐known factors like the growth hormone or estrogen, whose level declines with age and contributes to impaired bone formation, hardly any other circulating factors are known in this context (Worley, 1981; Frindik *et al*., 1999). We hypothesized that such factors might be provided by microvesicles (MVs) that are known to be contained in most body fluids (Mathivanan *et al*., 2010). Recently, circulating MVs were not only shown to stabilize and protect their cargo efficiently from degradation, but also to contribute to a directed and highly specific cell–cell communication (Mathivanan *et al*., 2010; Ekstrom *et al*., 2012).

As potential source of factors impacting on stem cell fate, we focused on the microvesicular secretome of endothelial cells (ECs) for several reasons: (i) ECs line the vasculature and are therefore able to secrete factors into the circulation; (ii) senescent ECs that were shown to exhibit an altered intracellular expression profile compared with early passage quiescent cells *in vitro* were also observed *in vivo* (Erusalimsky & Kurz, 2006 *;* Minamino & Komuro, 2007; Erusalimsky, 2009); (iii) MSCs are in close proximity to ECs within the bone (Shi & Gronthos, 2003; Crisan *et al*., 2008). In such microvesicles derived from senescent ECs, we found that miR‐31 is enriched within secreted CD63‐positive MVs of replicatively and stress‐induced senescent ECs and that miR‐31 transferred via microvesicles is conferring the osteogenesis‐inhibiting activity of senescent cell‐derived microvesicles. These findings demonstrate that not only the transcription of miRNAs (Hackl *et al*., 2010) but also their secretion changes when cells enter senescence. That this *in vitro* observation might also be relevant *in vivo* is supported by many publications which report on alterations in the level and composition of circulating miRNAs associated with various health problems, such as tumors or age‐associated diseases (Chen *et al*., 2008; Weilner *et al*., 2012; Noren Hooten *et al*., 2013; Olivieri *et al*., 2013). But how many microvesicles in the circulation might derive from the endothelium? Current estimates suggest that up to 8% of circulating microvesicles are derived from ECs in young mice (Hunter *et al*., 2008) and humans (Diehl *et al*., 2012). However, the amount of microvesicles released by ECs might be larger than initially expected due to sampling artifacts. In addition, the number of microvesicles secreted by senescent cells (Lehmann *et al*., 2008) is higher than from early passage cells, which might be caused by p53 activation (Yu *et al*., 2006). This would also suggest that in elderly organisms, the relative amount of microvesicles derived from senescent ECs might be even higher. Due to these data indicating the *in vivo* relevance of circulating microvesicles, we tested whether miR‐31 high plasma levels are also found in elderly individuals. Indeed, miR‐31 turned out to be upregulated in elderly donors, as well as in patients suffering from osteoporosis. Furthermore, our data show that vesicular miR‐31 is contributing to the osteogenesis‐inhibiting property of microvesicles isolated from plasma of elderly donors.

The observation of this study that anti‐miR‐31 transfection of ASCs alone had no effect on osteogenesis, explainable by the observation that ASCs exhibited relatively low intracellular miR‐31 levels, but that transfection of ASCs exposed to MVs had a positive effect on osteoblastogenesis, emphasizes once more the importance of the stem cell environment.

miR‐31 turned out as a master regulator of osteogenesis recently. Specifically, its inhibition by anti‐miR‐31 in bone marrow stromal stem cells seeded onto a scaffold in a critical‐sized calvarial defect in rats leads to more new bone formation compared with nontargeting control‐transfected cells (Deng *et al*., 2014). This effect might be mediated by repression of its already known osteogenesis‐relevant targets Runx2 (Deng *et al*., 2013a, 2013b), Osterix (Baglio *et al*., 2013), and SATB2 (Deng *et al*., 2013a, 2013b), to which we here add FZD3 mRNA, a validated target of miR‐31 in the field of breast cancer metastasis (Valastyan *et al*., 2009) and a member of the noncanonical Wnt signaling pathway (Lee *et al*., 2008). As FZD3 is robustly upregulated 4 days after induction of osteogenic differentiation, it might well turn out as a novel early marker of osteogenesis, as it was also found to increase during osteogenesis by other studies (Chakravorty *et al*., 2014).

Taken together, these data support our hypothesis that decreased osteogenesis *in vivo* might be caused by secreted and circulating microvesicles high in miR‐31. Their uptake into MSCs might then lead to impaired osteoblastogenesis which in turn might shift the delicate balance between bone‐forming osteoblasts and bone‐resorbing osteoclasts to the osteoclast resulting in reduced bone mineral density and bone quality (Fig. 4L).

Summarizing, we show for the first time that the microRNA content of microvesicles from senescent cells contributes to an aged systemic environment that fails to support tissue regeneration. Thus, miR‐31 might represent a biomarker and therapeutic target for conditions/diseases whenever osteoblast differentiation is a limiting factor. Finally, we also suggest that understanding and controlling the systemic environment might turn out to be a key factor for the success of stem cell‐based medicine, especially in regard to age‐associated diseases.

## Experimental procedures

Experimental procedures are described in detail in the supplements.

## Funding

This work has been supported by FWF Project P24498, the GEN‐AU Project 820982 ‘Non‐coding RNAs’, FP7 project FRAILOMIC, as well as grants by the Herzfelder'sche Familienstiftung. The financial support by the Austrian Federal Ministry of Economy, Family and Youth and the National Foundation for Research, Technology and Development is gratefully acknowledged as well as financial support by Chanel Research and Technology.

## Conflict of interest

JG and RGV are cofounders of Evercyte GmbH, Vienna, Austria, and of TAmiRNA GmbH, Vienna, Austria. A patent application describing miR‐31 as a marker and therapeutic target in deranged bone metabolism has been filed.

## Author contribution

SW and ES planned and performed the experiments, wrote the manuscript, and interpreted the results. MW planned the experiments and interpreted the data. PM planned and performed electron microscopy and interpreted the data. KS, KW, and KF performed the experiments. LM provided ideas and reagents. ABM, RW, and HR planned and provided serum samples. SuWo, HR, and PP planned and provided mesenchymal stem cells. PJ‐D contributed to planning and discussion. RG‐V contributed to planning and writing. JG contributed to planning, interpreted the results, and wrote the manuscript.

## Supporting information


**Fig. S1** (A) Representative microscope image of ASCs showing a typical morphology. (B) Representative flow cytometric analysis of two stem cell donors used in this study, ASC57 and ASC60, show expression of ASC specific (ASC*) and typical mesenchymal stem cell surface markers and did not show hematopoietic stem cells surface marker. (C) Early quiescent passage (PD13) and senescent (PD53) endothelial cells that were used for isolating MVs from conditioned medium were stained for senescence‐associated β‐galactosidase activity, representative images using the same magnification are shown.

**Fig. S2** (A) SIPS treatment of endothelial cells results in growth arrest at 75 μm tBHP**.** HUVECs were pulsed with the indicated concentration of tBHP. Growth was monitored by cell counting. Representative figures until day 5 of treatment indicating growth arrest at the highest dose are shown. MVs were isolated from tBHP pulsed endothelial cells after 14 days, when no additional cell proliferation was observed in the cells treated with 75 μm tBHP. In contrast, after exposure to 35 μm tBHP the cells completely recovered and resumed growth (*N *= 3). miR‐31 is also induced by tBHP treatment of HUVECs (B) endogenously and (C) accumulates in MVs derived from treated versus replicative senescent or untreated HUVECs. Error bars indicate the standard deviations of 3 independent measurements. miR‐31 is upregulated intracellularly in senescent (S) versus early quiescent passage (Y) (D) human liver endothelial cells (ECs) (*N *= 3) and (E) human retinal microvascular endothelial cells (hReEC) (*N *= 3) as analyzed by qPCR. *: *P* < 0.05, **: *P* < 0.01, ***: *P* < 0.001. Data are presented as mean values ± SD.

**Fig. S3** (A) QPCR showing increased intracellular miR‐31 levels 24 h and 48 h after transient transfection of ASCs compared with nontargeting miRNA control (miRC) or MOCK‐transfected (untransf.) cells. (B, C) Confirmation that miR‐31 is a general regulator of osteogenesis. Reduced osteogenic differentiation capacity of (B) C3Ht101/2 and (C) C2C12 cells, two mouse mesenchymal mulitpotent cell lines, by transient transfection of miR‐31 compared with nontargeting miRNA control (miRC) and MOCK‐transfected (untransf.) cells measured by Osteocalcin reporter activity. (*N *= 4). (D) Confirmation of FZD3 knockdown of siFZD3‐transfected cells compared with nontargeting miRNA control (miRC)‐transfected ASCs using qPCR. (E) Taqman based qPCR showing downregulated intracellular miR‐31 levels 24 h after transient anti‐miR‐31 transfection compared with nontargeting miRNA control (anti‐miRC)‐transfected cells.). ns: not significant, *: *P* < 0.05, **: *P* < 0.01, ***: *P* < 0.001. Data are presented as mean values ± SD.

**Data S1** Experimental procedures.
