# Supplementary figures and images for: Secreted microvesicular miR‐31 inhibits osteogenic differentiation of mesenchymal stem cells
Source: Aging Cell. 2016 May 4;15(4):744–54. doi: 10.1111/acel.12484 (PMC4933673; doi:10.1111/acel.12484)

Figure S1

A

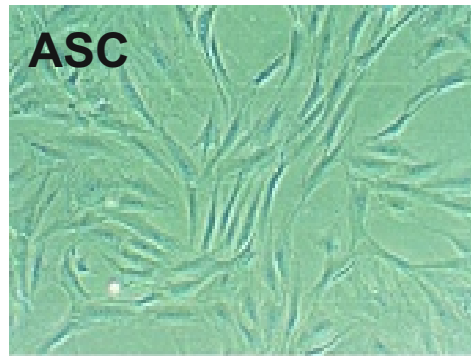

B

|         | ASC57 | ASC60 | ASC* |
|---------|-------|-------|------|
| CD13    | +     | +     | +    |
| CD14    | -     | -     | -    |
| CD34    | -     | -     | -    |
| CD45    | -     | -     | -    |
| CD73    | +     | +     | +    |
| CD90    | +     | +     | +    |
| HLA-ABC | +     | +     | +    |
| HLA-DR  | -     | -     | -    |

C

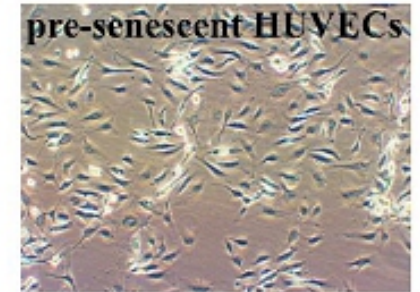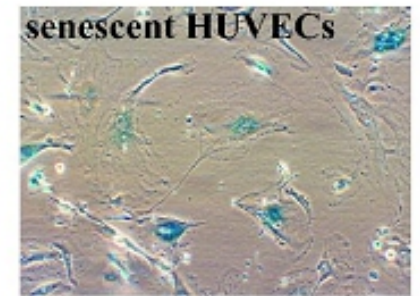

Supplement: Supplementary file 1 — Fig. S1 (A) Representative microscope image of ASCs showing a typical morphology. (B) Representative flow cytometric analysis of two stem cell donors used in this study, ASC57 and ASC60, show expression of ASC specific (ASC*) and typical mesenchymal stem cell surface markers and did not show hematopoietic stem cells surface marker. (C) Early quiescent passage (PD13) and senescent (PD53) endothelial cells that were used for isolating MVs from conditioned medium were stained for senescence‐associated β‐galactosidase activity, representative images using the same magnification are shown. [file ACEL-15-744-s001.pdf]

Figure S2

D

human liver ECs

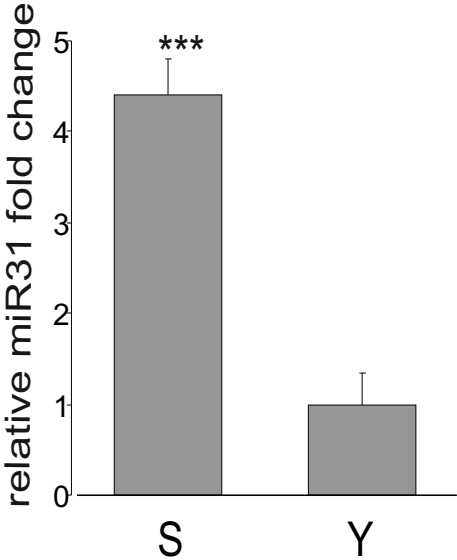

E

hReEC

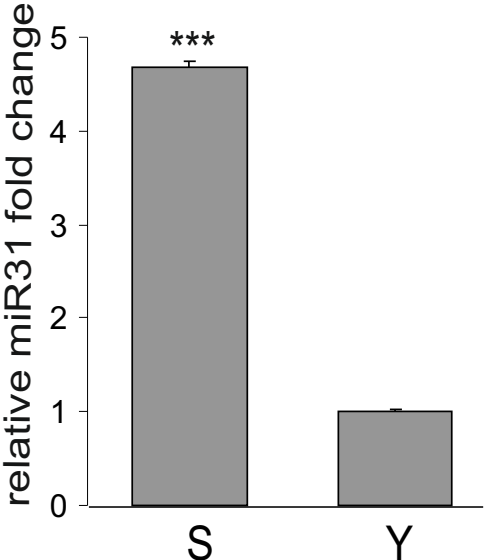

Figure S2

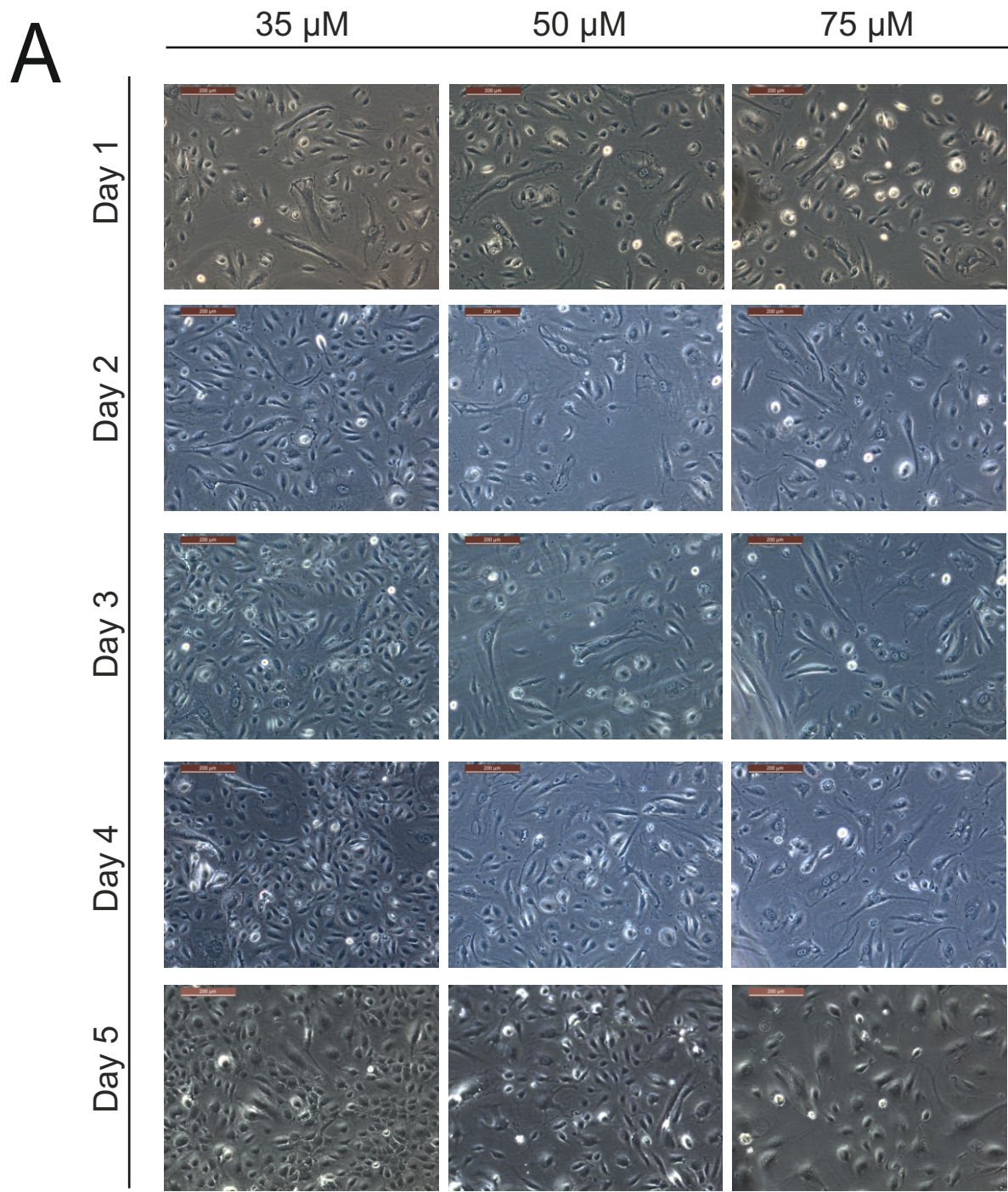

Figure S2

B

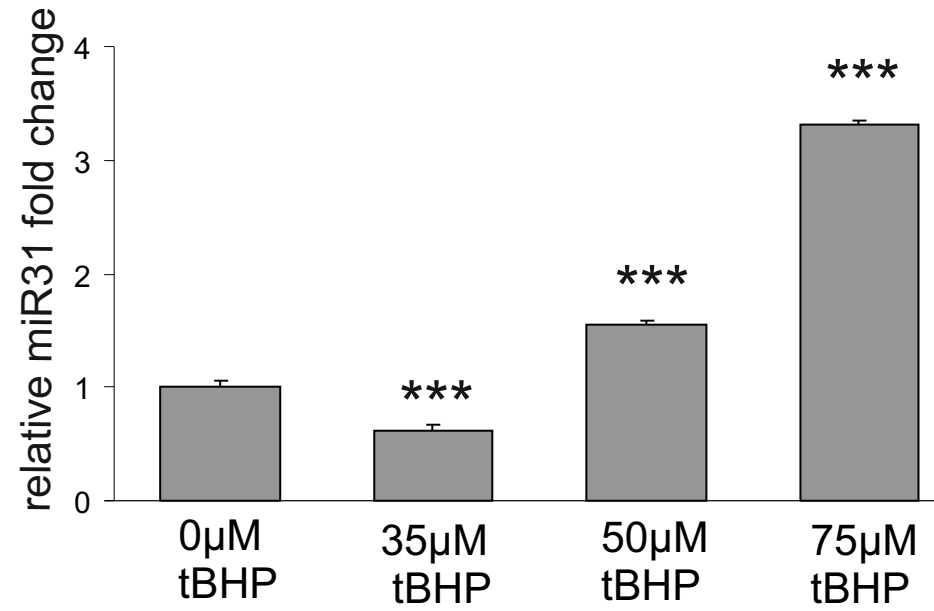

C

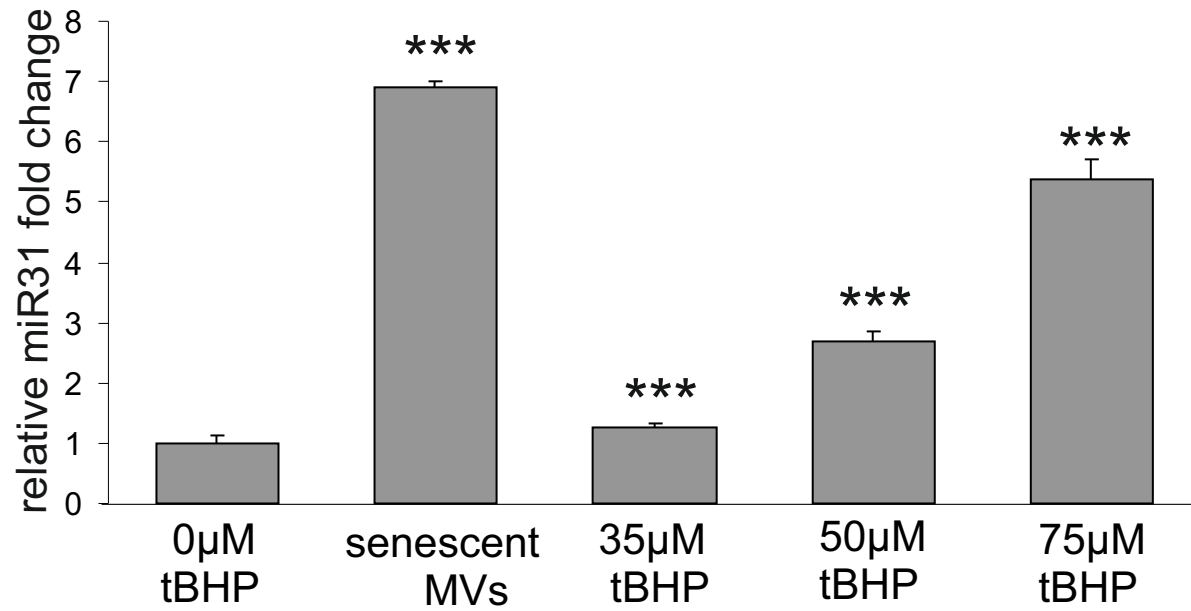

Supplement: Supplementary file 2 — Fig. S2 (A) SIPS treatment of endothelial cells results in growth arrest at 75 μm tBHP. HUVECs were pulsed with the indicated concentration of tBHP. Growth was monitored by cell counting. Representative figures until day 5 of treatment indicating growth arrest at the highest dose are shown. MVs were isolated from tBHP pulsed endothelial cells after 14 days, when no additional cell proliferation was observed in the cells treated with 75 μm tBHP. In contrast, after exposure to 35 μm tBHP the cells completely recovered and resumed growth (N = 3). miR‐31 is also induced by tBHP treatment of HUVECs (B) endogenously and (C) accumulates in MVs derived from treated versus replicative senescent or untreated HUVECs. Error bars indicate the standard deviations of 3 independent measurements. miR‐31 is upregulated intracellularly in senescent (S) versus early quiescent passage (Y) (D) human liver endothelial cells (ECs) (N = 3) and (E) human retinal microvascular endothelial cells (hReEC) (N = 3) as analyzed by qPCR. *: P < 0.05, **: P < 0.01, ***: P < 0.001. Data are presented as mean values ± SD. [file ACEL-15-744-s002.pdf]

Figure S3

A

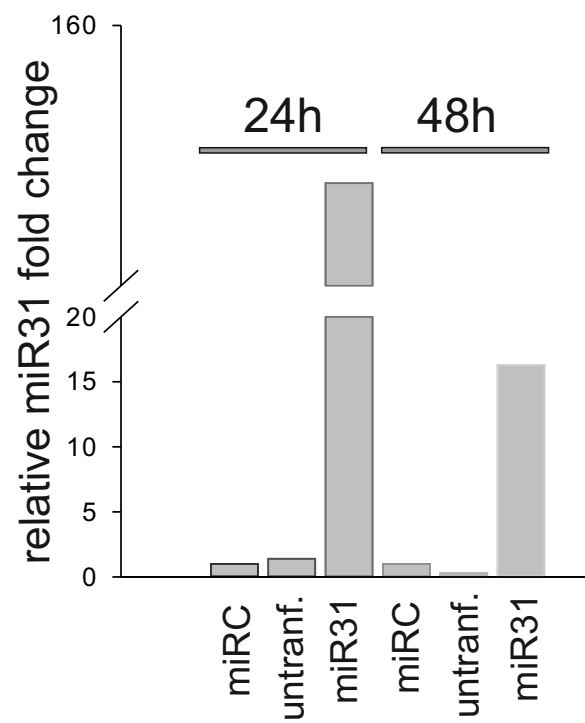

B

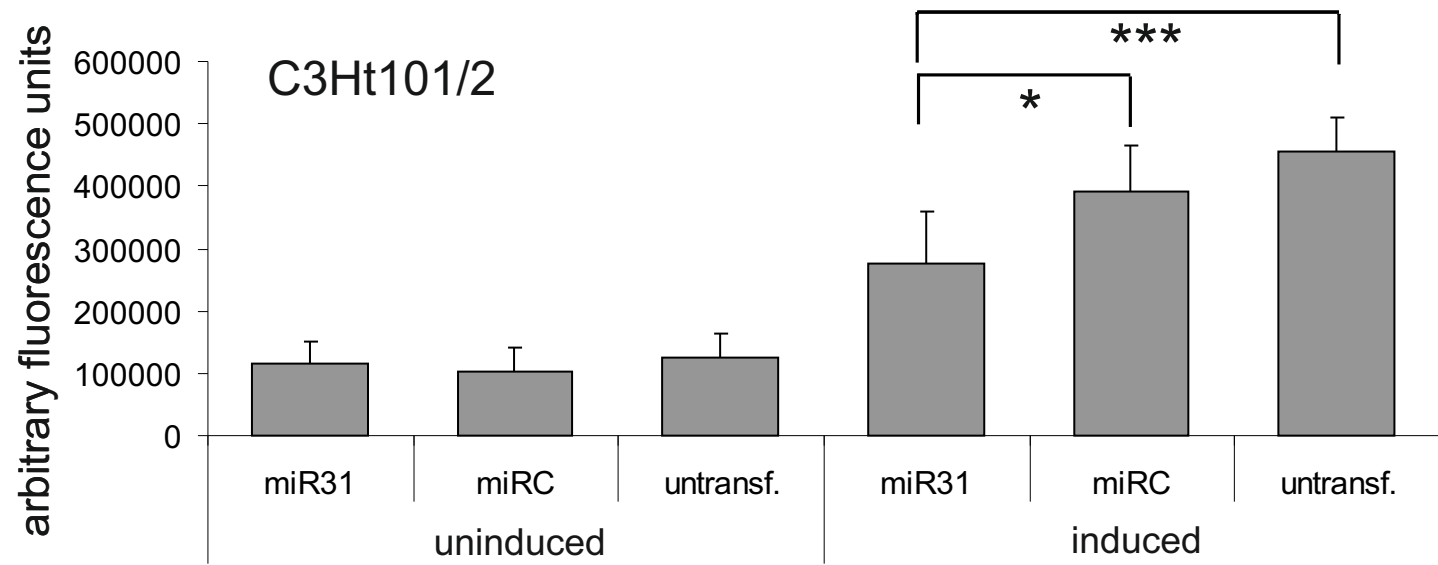

# Figure S3

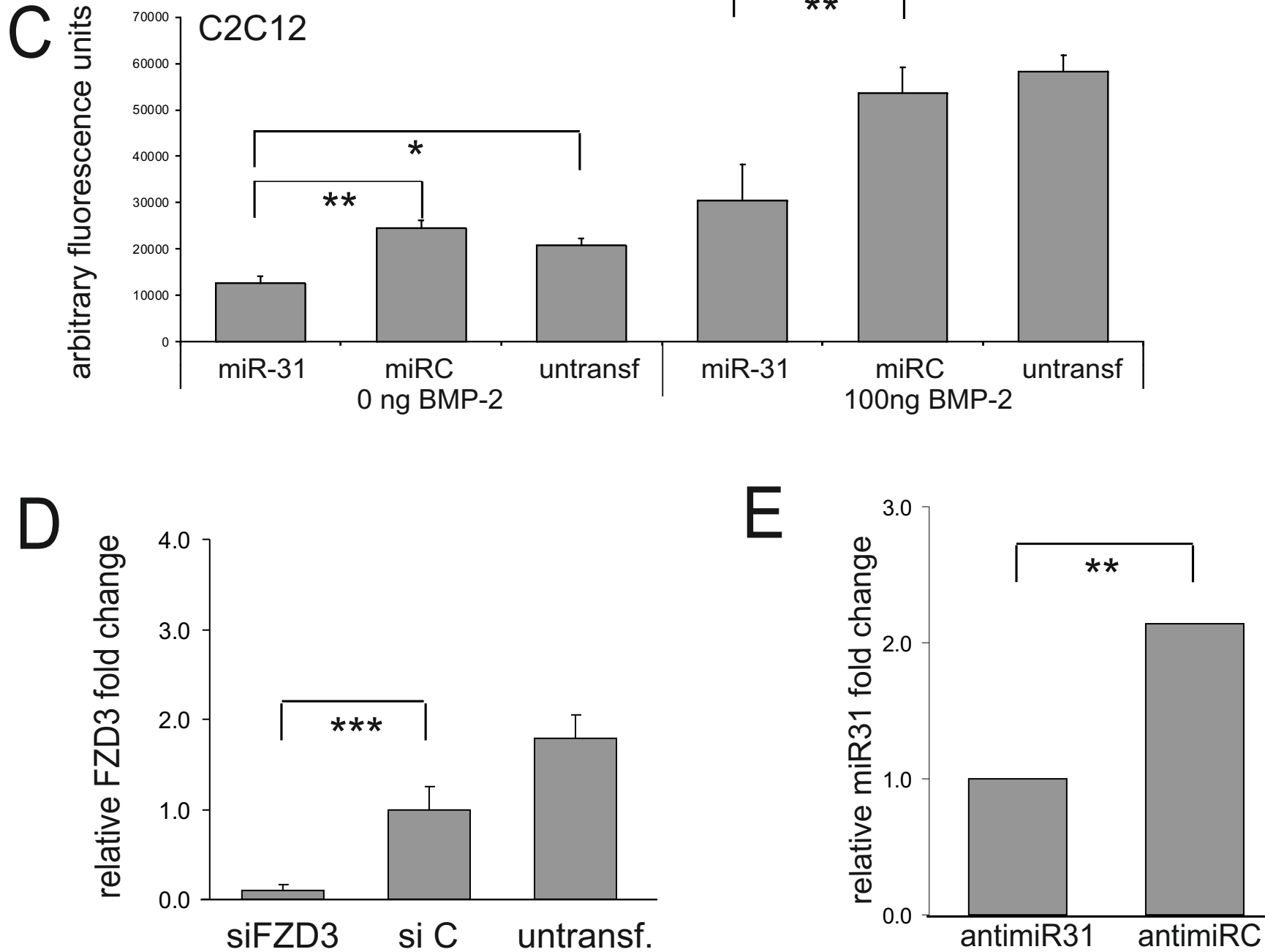

Supplement: Supplementary file 3 — Fig. S3 (A) QPCR showing increased intracellular miR‐31 levels 24 h and 48 h after transient transfection of ASCs compared with nontargeting miRNA control (miRC) or MOCK‐transfected (untransf.) cells. (B, C) Confirmation that miR‐31 is a general regulator of osteogenesis. Reduced osteogenic differentiation capacity of (B) C3Ht101/2 and (C) C2C12 cells, two mouse mesenchymal mulitpotent cell lines, by transient transfection of miR‐31 compared with nontargeting miRNA control (miRC) and MOCK‐transfected (untransf.) cells measured by Osteocalcin reporter activity. (N = 4). (D) Confirmation of FZD3 knockdown of siFZD3‐transfected cells compared with nontargeting miRNA control (miRC)‐transfected ASCs using qPCR. (E) Taqman based qPCR showing downregulated intracellular miR‐31 levels 24 h after transient anti‐miR‐31 transfection compared with nontargeting miRNA control (anti‐miRC)‐transfected cells.). ns: not significant, *: P < 0.05, **: P < 0.01, ***: P < 0.001. Data are presented as mean values ± SD. [file ACEL-15-744-s003.pdf]
