# Supplementary material for: Secreted microvesicular miR‐31 inhibits osteogenic differentiation of mesenchymal stem cells
Source: Aging Cell. 2016 May 4;15(4):744–54. doi: 10.1111/acel.12484 (PMC4933673; doi:10.1111/acel.12484)
Supplement: Supplementary file 4 — Data S1 Experimental procedures. [file ACEL-15-744-s004.docx]

Experimental procedures

Cell culture

Human umbilical vein endothelial cell (HUVEC)

Endothelial cells were isolated from human umbilical veins as described ^1,2^. HUVECs were cultivated in gelatin pre-coated flasks in M199 with Earle's salts supplemented with 4mM glutamine, 15% fetal calf serum (FCS) and 10% endothelial cell growth supplement (ECGS) containing 170 U/ml heparin at 37°C in a humidified atmosphere with 5% CO_2_. Cells were passaged once or twice a week at a split ratio of 1:2 to 1:4 according to the growth rate. HUVECs were cultivated to senescence and stained for senescence associated β-galactosidase (SA-β-gal) activity as described previously (Chang et al., 2005). For collection of supernatants, contact inhibited (quiescent, PD21) and senescent (PD49 / 95% SA-β-gal positive) cells were allowed to secrete into ASC or HUVEC medium, depending on the experiment, for 48 hours.

SIPS was induced by tBHP treatment of HUVECs on 5 consecutive days for one hour each by adding tBHP to a final concentration of 35 μM, 50 μM or 75 μM in the medium. Permanent growth arrest was induced by 75 μM tBHP as assessed by microscopic follow up for 14 days. Supernatants were collected, centrifuged at 1900g, 4°C and used freshly for MV preparation or stored at -80°C. Supernatant volumes were normalized to the number of HUVECs at the time of supernatant harvest. Cell culture medium incubated for 48 hours at 37°C was used as additional control.

Human adipose-derived stem cells (ASCs)

Subcutaneous adipose tissue was obtained during outpatient tumescence liposuction under local anesthesia with patient consent. ASCs were isolated as described before^3,4^ and cultured in DMEM-low glucose/HAM´s F-12 supplemented with 4mM L-glutamine, 10% fetal calf serum (FCS, PAA) and 1ng/mL recombinant human basic fibroblast growth factor (rhFGF, R&D Systems) at 37°C, 5% CO_2_ and 95% air humidity. Cells were passaged once or twice a week at a split ratio of 1:2 according to the growth rate.

All differentiation protocols were carried out in 24 well cell culture plates. For osteogenic differentiation ASCs were seeded at a density of 2x10^3^ cell per well. 72 hours after seeding cells were incubated with osteogenic differentiation medium (DMEM-low glucose, 10% FCS, 4mM L-glutamine, 10nM dexamethasone, 150μM ascorbate-2-phosphat, 10mM β-glycerolphosphate and 10nM vitamine-D3) up to 4 weeks.

For Alizarin staining of calcified structures, cells were fixed for 1 hour in 70% ethanol at -20°C. After brief rinsing, cells were stained for 20 minutes with 40mM Alizarin Red solution (Sigma) and washed with PBS. For quantification Alizarin was extracted for 30 minutes using 200μl 0.1M HCL/0.5% SDS solution. The extracted dye was measured at 425nm.

C2C12 and C3Ht101/2

The mouse skeletal osteo/myoblast cell line (C2C12) obtained from ATCC were cultured in Dulbecco’s Modified Eagle’s Medium (DMEM) high glucose, supplemented with 5% heat inactivated fetal calf serum (FCS) and 2mmol/L L-glutamine at 37°C in a humidified atmosphere with 5% CO2.

Mouse embryonic fibroblast cells C3Ht10 1/2 obtained from ATCC were cultured in DMEM/Ham´s F-12 containing 10% FCS at 37°C in a humidified atmosphere with 5% CO_2_.

The influence of miRNA-31 transfection on osteogenic differentiation was analyzed using the C2C12 and C3Ht10 1/2 cell line in conjunction with an osteocalcin specific reporter gene assay^5^. C2C12 and C3Ht10 1/2 cells are capable of differentiating to the osteogenic lineage upon treatment with recombinant BMPs, which is observable by the induction of alkaline phosphatase, osteocalcin and other osteoblast specific genes. The cells, seeded in a T175, were first transfected with 99µg of the osteocalcin reporter system and then 24 h post reverse transfected with 30nM miRNA-31, antimiR-31 or scrambled miRNA control #2 as control using Ambions siPORT^TM^ *Neo*FX^TM^. Osteogenic differentiation was induced using 100ng/ml or 300ng/ml recombinant BMP2 (InductOS, Pfizer), controls were not induced with growth factor. Osteocalcin reporter activity was assessed after 6 days of differentiation by determining Metridia luciferase activity in the cell culture supernatants using Clontech Ready-To-Glow Secreted Luciferase System Kit. On day 12 Alizarin red s staining was performed to further analyze the degree of differentiation as described above.

Transfections

ASCs were transfected using siPORT^TM^ *Neo*FX^TM^ transfection reagent (Applied Biosystems). Cells were transfected with 10nM precursor hsa-miR-31, antimiR-31, celmiR-39 or scrambled miRNA control #2 (Ambion), as well as with 10nM FZD3 siRNA (Dharmacon, L-005502-00-0005) or the corresponding control (Dharmacon) according to the manufacturer’s protocol. Three days after transfection, differentiation was started as described before.

HUVECs were transfected by the use of Neon transfection system (Thermo scientific) as previously described^6^. Cells were transfected with celmiR-39 or scrambled miRNA control #2 (Ambion), as well as with ON-TARGETplus Human CD63 (967) siRNA (Dharmacon, L-017256-00-0005) or the corresponding control (Dharmacon) according to the manufacturer’s protocol. Two days after transfection, MVs were isolated or proteinlysates were prepared.

Assessment of apoptotic cell death

HUVECs were seeded in 12-well cell culture plates and were allowed to secrete into ASC or HUVEC medium for 48 hours. Thereafter, the cells were detached using 50mM EDTA and stained with Annexin V- FITC and PI (Roche) according to the manufacturer´s instructions. Analysis of the percentage of apoptotic and necrotic/late-apoptotic cells were performed using a FACS-Calibur and the CellQuest software (Becton Dickinson).

Quantitative real-time PCR

Osteogenic differentiation capacity was additionally confirmed by performing quantitative real-time PCR (qPCR) on various osteogenic differentiation marker genes. Therefore total ASCs RNA was isolated using Trizol (Invitrogen) at different time points before and during osteogenesis. 4 days after differentiation start the marker Frizzled-3 (FZD3) was used, 7 days after differentiation start the early osteogenic marker alkaline phosphatase (ALP) was analyzed and 21 days after differentiation start the late osteogenic marker osteocalcin (OC) was tested. qPCRs on the intracellular osteogenic marker were normalized to GAPDH. Reverse transcription was performed using DyNAmo cDNA Synthesis Kit (Biozym) and qPCR was performed using the RotorGene2000 (Corbett).

For miRNA analysis specific TaqMan assays (Applied Biosystems) were used according to manufactures protocol. Intracellular miRNA levels were normalized to the snRNA U6 as internal control, while the number of secreting cell as well as spiked in cel-miR39 as reference, were used in order to compare secreted miRNA levels.

In order to test for CD63 Antibody specificity, qPCR on CD63 of siRNA against CD63 transfected HUVECs was performed and normalized to GAPDH. Reverse transcription was performed using DyNAmo cDNA Synthesis Kit (Biozym) and qPCR was performed using the RotorGene2000 (Corbett).

For isolation of RNA from blood samples, 250-500μl serum was used to isolate total RNA using Trizol LS reagent (Invitrogen). To allow for normalization of sample-to-sample variation in RNA isolation, 25fmol synthetic *C. elegans* miRNAs cel-miR-39 were added before isolation. Serum samples (12 healthy old donors and 17 healthy young donors) were obtained from R. Westendorp, Department of Gerontology & Geriatrics C2-R, Leiden University Medical Center, The Netherlands or C. Gabriel, Blutzentrale, Linz, Austria. Osteoporotic serum samples were obtained from Heinrich Resch, Department of Medicine 2, St. Vincent Hospital, Vienna, Austria. Institutional ethics committees approved the study, and written, informed consent has been obtained from each subject.

Microvesicle (MV) purification

MV were purified by filtration and differential centrifugation as described previously^7^. In brief, supernatants were collected after incubation of 48 hours. This conditioned media were centrifuged at 500g for 10 minutes to sediment cells and at 14.000g for 15 minutes to eliminate cell debris and filtered through a 0.22μm filter excluding a fraction of apoptotic bodies. MVs were then pelleted by ultracentifugation at 100.000g for 60 minutes and the resultant pellet was washed with PBS. MV were used as fresh preparations for electron microscopy or conserved at -80°C for further analysis. For differentiation studies MV derived from 2x10^4^ HUVECs or 1 ml of human serum were resuspended in 50μl PBS and added per well ASCs.

Purification of CD63-positive microvesicles

Preparation of Immunoaffinity Capture Microbeads

CD63 monoclonal antibody immunoaffinity capture microbeads (Dynabeads^®^ M-270 Epoxy, Invitrogen) were prepared using Dynabeads® Antibody Coupling Kit (Invitrogen) according to the manufacturer’s protocol. Briefly 5 mg of Dynabeads were washed with 1 ml of C1 solution. The supernatant was removed by magnetic sedimentation of the beads and 50µg of mouse monoclonal CD63 antibody (IgG1) (ab8219 Abcam) or the corresponding mouse IgG1 Isotype control (Thermo Scientific) were mixed with 200 µl of C1 solution. Washed beads were first mixed with prepared antibody solution and 250µl of C2 solution were added afterwards. Beads were incubated at 37°C on a roller overnight.

The next day supernatant was removed by placing the tube on a magnet whereby beads were able to collect at the tube wall. Afterwards beads were washed with each 800µl of HB, LB and finally SB buffer and stored at 4°C until use.

Depletion of CD63-positive microvesicles by immunoaffinity capture microbeads

Isolated MVs were resuspended in PBS. The MV-solution was split and incubated either with CD63 or with IgG1 Isotype control antibody coupled Dynabeads for 2 h at 4°C on a roller. Afterwards the tube was placed on a magnet allowing the beads to collect at the wall. Supernatant containing MV depleted of CD63-positive microvesicles was decanted. MV depleted of CD63-positive microvesicles or MV exposed to the control Antibody-coupled beads derived from either 2x10^4^ HUVECs or 1 ml of human serum were resuspended in 50μl PBS and added per well ASCs.

Electron microscopy

Purified MVs were left to absorb on coated nickel grids (200 mesh, hexagonal, Pioloform-coated Athene nickel grids). After fixation with 4% paraformaldehyd MVs were stained with 2% uranyl acetate for 30 seconds, the grids were left to dry and the absorbed material was visualized using transmission electron microscopy (TEM), (Philips model CM 12 electron microscope, Philips, Eindhoven, NL).

For electron microscopy *in-situ* hybridization (EM-ISH) MV pellets were permeabilized with 0.1% Triton-X for 5 minutes at room temperature. After washing with PBS MVs were incubated for at least 4 hours with hybridization buffer as described previously^8^. For each sample 1pM of the LNA DIG-labelled single stranded probe (Exiqon, Denmark) was denaturated in denaturizing hybridization buffer (containing 50% formamide, 5x SSC, 5x Denhardt´s solution, 0.1% Tween, 0.25% CHAPS, 200μg ml^-1^ yeast RNA, 500μg ml^-1^ salmon sperm DNA) by incubation at 80°C for 5 minutes. Probes were placed on ice quickly. MV were mixed with the probe and hybridized at 50°C overnight. After hybridization samples were washed stringently with 0.2 x SSC at 60°C for 1 hour. Subsequently, MVs were incubated with Anti-DIG antibody (Roche) for 30 minutes and an additional hour with the second 5nm gold particle labelled antibody (Sigma). After washing with PBS MV were embedded in Epon, and 80 nm, on average, sections were cut using an ultramicrotom (Ultracut, Reichert) and then analyzed by transmission electron microscopy (TEM, Philips model CM 12 electron microscope ,Philips, Eindhoven, NL).

Western Blot

Total proteins and proteins from microvesicles, as well as the fraction depleted of CD63-positive microvesicles were extracted and separated on polyacrylamide gels, before transfer to a PVDF membrane (Roth, Germany). The membrane was blocked in 3% skimmed milk, incubated with the CD63 antibody (10628D Thermo Scientific). Alexa Fluor 680-conjugated anti-mouse IgG (Molecular Probes) was used as secondary antibody. Signal intensities were analyzed by using the Odyssey infrared image system (LiCor) respectively.

Osteoporosis Patients

14 men with idiopathic osteoporosis and a mean age ~53 years were studied. Osteoporosis was defined by the presence of low trauma fractures at vertebral or peripheral sites and/or by dual energy X rax absorptiometry (T‑scores less than ‑ 2.5). In all subjects secondary causes of osteoporosis (such as glucocorticoid treatment, alcohol abuse or thyrotoxicosis) had been excluded by an appropriate clinical and laboratory evaluation.

Statistics

Data were statistically analyzed using Student´s *t* test, one-way ANOVA and one-way ANOVA followed by the Dunn´s method as indicated. Analyses were performed with SigmaPlot 10.0 (SigmaPlot, Germany). The tests were two-sided with type 1 error probability of 0.05. Data are presented as mean values ± SD.

Samples were not blinded systematically, but repetitions of experiments were performed by different operators and methods having unbiased read-outs were chosen.

References

1. Chang, M.W., et al. Comparison of early passage, senescent and hTERT

immortalized endothelial cells. Experimental cell research 309, 121-136

(2005).

1. Jaffe, E.A., Nachman, R.L., Becker, C.G. & Minick, C.R. Culture of human

endothelial cells derived from umbilical veins. Identification by morphologic

and immunologic criteria. *The Journal of clinical investigation* **52**, 2745-2756

(1973)

1. Wolbank, S.*, et al.* Dose-dependent immunomodulatory effect of human stem

cells from amniotic membrane: a comparison with human mesenchymal stem

cells from adipose tissue. *Tissue engineering* **13**, 1173-1183 (2007).

1. Wolbank, S.*, et al.* Telomerase Immortalized Human Amnion- and Adipose-

Derived Mesenchymal Stem Cells: Maintenance of Differentiation and

Immunomodulatory Characteristics. *Tissue Eng Part A* (2009).

1. Feichtinger, G.A.*, et al.* Enhanced reporter gene assay for the detection of osteogenic differentiation. *Tissue engineering. Part C, Methods* **17**, 401-410 (2011).
2. Weilner, S, et al. Vesicular Galectin-3 levels decrease with donor age and contribute to the reduced osteo-inductive potential of human plasma derived extracellular vesicles. Aging, Vol. 8 No.1 (2016)
3. Lehmann, B.D., et al. Senescence-associated exosome release from human prostate cancer cells. Cancer research 68, 7864-7871 (2008).
4. Obernosterer, G., Martinez, J. & Alenius, M. Locked nucleic acid-based in situ detection of microRNAs in mouse tissue sections. Nature protocols 2, 1508-1514 (2007).
